# Neurophysiological and autonomic patterns associated with heartfulness and bell meditation: a three-arm exploratory study

**DOI:** 10.3389/fpsyg.2026.1787149

**Published:** 2026-04-28

**Authors:** Sushil Prasad Mahato, M. Anjaladevi, Abinash Roy, Pailoor Subramanya, Samiran Mondal

**Affiliations:** 1 Department of Yoga Studies, School of Medicine and Public Health, Central University of Kerala, Kasaragod, India; 2 Department of Yogic Art & Science, Vinaya Bhavana (Institute of Education), Visva-Bharati University, Santiniketan, India

**Keywords:** bell meditation, brain–body integration, electroencephalography (EEG), gamma amplitude, heartfulness meditation, heart-rate variability (HRV), low-beta activity

## Abstract

Meditation facilitates brain–body integration by synchronizing neural oscillations with autonomic rhythms. Nonetheless, the effects of heartfulness (heart-centered) and bell (sound-based) meditation on electroencephalography (EEG) and autonomic markers remain insufficiently explored. In this randomized, exploratory pilot study, nine healthy male university students were allocated to the heartfulness meditation group (HMG; *n* = 3), bell meditation group (BMG; *n* = 3), or control group (CG; *n* = 3) for 21 days (30 min per session, 5 days per week). Resting EEG gamma and low-beta activity (12–15 Hz), blood volume pulse (BVP)-derived heart rate (HR), and heart-rate variability (HRV) indices, including low-frequency percentage (LF%) and HRV amplitude, were recorded both pre- and post-intervention. The analyses were descriptive and summarized using Hedges’ g and 95% confidence intervals (CIs). The HMG exhibited exhibited observed increases in gamma and low-beta (g ≈ 1.0–2.3), the BMG demonstrated a very observed low-beta activity increase with a small gamma change, and the CG showed minimal change in both parameters. HR decreased across all groups, LF% declined in the HMG and BMG but increased in the CG, and HRV amplitude decreased in all groups. These preliminary observations indicate a potential trend toward neural–autonomic regulation, with heartfulness emphasizing attentional engagement and bell emphasizing relaxation. The limitations include the very small, male-only sample, brief duration, and reliance on BVP-derived HRV. Larger, adequately powered trials with more comprehensive EEG/HRV metrics and behavioral outcomes are required to confirm these findings.

## 1 Introduction

Meditation refers to the intentional cultivation of mindfulness or directed focus on tangible or intangible objects (Roy and Subramanya, 2025). Contemporary research increasingly situates meditation within the framework of brain–body integration, highlighting how meditative states align neural oscillations with autonomic rhythms to enhance cognitive function, emotional stability, self-regulation, and overall well-being (Steinhubl et al., 2015). Neural–cardiac coupling, defined as the dynamic interaction between cortical processes and autonomic cardiac control, is commonly assessed using simultaneous neural (EEG, fMRI) and cardiac (heart rate, HRV) measures. This bidirectional loop reflects the top-down influences of cortical oscillations on autonomic tone and bottom-up vagal afferents shaping cognition and affect through neural–autonomic interplay (Dalton et al., 2005; Machetanz et al., 2021). Enhanced parallel physiological shifts between EEG activity and HRV is considered a psychophysiological marker of balanced arousal and resilience, providing an empirical index linking brain function, autonomic health, and psychological wellbeing.

A growing body of evidence shows that meditation enhances synchrony between medium-to-high-frequency EEG rhythms (e.g., alpha and gamma) and parasympathetic HRV indices (HF power), suggesting improved autonomic and cognitive-affective regulation (Ulke et al., 2017; Kim et al., 2013). Gamma oscillations (~30–80 Hz) have been linked to neuroplastic adaptations in attentional networks and are associated with heightened attention, with increases observed in both long-term practitioners and novices following brief training (Braboszcz et al., 2017). Mindfulness practices also enhance low-beta activity activity, which supports sensorimotor integration, motor planning, and attentional control (Brown et al., 2021). Parallel findings in autonomic physiology demonstrate that HRV, a key marker of parasympathetic regulation, increases reliably during meditation, reflecting improved vagal tone and autonomic balance (Brown et al., 2021). These changes correspond to enhanced emotional regulation, greater stress resilience, reduced sympathetic arousal, and improved cardiovascular health, establishing HRV as a biomarker of the benefits of meditation (Krygier et al., 2013).

Despite extensive research on mindfulness, Zen, and transcendental meditation, heartfulness meditation (HM), a modern, heart-centered practice emphasizing thoughtfulness and inner development—remains underexplored in terms of EEG and HRV outcomes, with most studies limited to experienced practitioners (Krishna et al., 2022). Similarly, bell meditation, which uses auditory cues such as periodic bells or Tibetan singing bowls to anchor attention (Burtner et al., 2002), has ancient roots but little systematic neurophysiological evaluation has been conducted. Comparative data across these distinct styles in healthy novices are rare, even though meditation type, duration, and expertise influence EEG and HRV profiles.

To address this gap, the present study conducted a randomized three-arm pre–post design comparing 21 days of heartfulness meditation, bell meditation, and a usual-routine control. EEG indices (gamma, Low-Beta Activity) and HRV parameters (HR, LF%, amplitude) were recorded before and after the intervention. Given its exploratory nature and very small sample size, the analyses were descriptive, focusing on individual trajectories and group-level change scores. This study provides parallel observations of neural and autonomic markers of heartfulness and bell meditation in novices, extending the comparative map of contemplative practices and providing a foundation for future adequately powered trials.

## 2 Methods

### 2.1 Study design and participants

This exploratory, three-arm, parallel-group randomized pre–post study included the heartfulness meditation group (HMG), bell meditation group (BMG), and control group (CG). Nine healthy male university students completed both baseline (pre) and post-intervention (post) assessments (*N* = 9; *n* = 3 per group; see Table 1). The study was approved by the Institutional Ethics Committee for Human Research, Visva-Bharati (Ref. No. VB/IECHR/03/2024), and all the participants provided written informed consent.

**Table 1:** Baseline characteristics of the participants (*n* = 9).

Characteristic	Heartfulness (*n* = 3)	Bell (*n* = 3)	Control (*n* = 3)
Age (years), Mean ± SD	19.7 ± 0.58	20.0 ± 1.00	19.3 ± 1.15
Gender, *n* (%)			
Male	3 (100%)	3 (100%)	3 (100%)
Height (cm), Mean ± SD	158.3 ± 6.1	157.0 ± 5.8	155.3 ± 6.7
Weight (kg), Mean ± SD	52.0 ± 5.0	55.3 ± 6.1	54.5 ± 5.5
BMI (kg/m^2^), Mean ± SD	20.7 ± 1.2	22.4 ± 1.6	22.1 ± 1.4
GHQ-12 score (Mean ± SD)	1.7 ± 1.0	1.3 ± 0.6	2.0 ± 1.2
Cleared PAR-Q, n (%)	3 (100%)	3 (100%)	3 (100%)
Educational level			
10 + 2	3 (100%)	3 (100%)	3 (100%)
Marital status			
Unmarried	3 (100%)	3 (100%)	3 (100%)
Socioeconomic status			
Lower	0	0	1 (33.3%)
Upper lower	1 (33.3%)	1 (33.3%)	0
Lower middle	2 (66.7%)	2 (66.7%)	2 (66.7%)
Upper middle/upper	0	0	0

Values are presented as mean ± SD or *n* (%). GHQ-12: 12-item General Health Questionnaire; PAR-Q: Physical Activity Readiness Questionnaire. No statistically significant between-group differences were observed at baseline (all descriptive *p* > 0.05).

### 2.2 Interventions

Over 21 days, the HGM group practiced silent, heart-focused meditation for 30 min/session, 5 days/week, guided by a certified trainer using a standardized protocol (Yadav et al., 2021). The BMG engaged in daily 30-min sound-based sessions using Tibetan singing bowl recordings with progressive tonal sequences, delivered according to a validated protocol (Goldsby et al., 2017). The CG continued their usual routine without any structured practice.

### 2.3 Recording procedure

Each participant underwent physiological recordings at two time points: baseline (Day 1), prior to the initiation of meditation practice, and post-intervention (Day 21), immediately following the final 30-min meditation session. The daily 30-min intervention sessions were conducted without any recording procedures. During both pre- and post-intervention assessments, participants were seated comfortably in a controlled environment and completed a continuous 10-min eyes-closed resting-state recording following an acclimatization period to ensure a stable physiological state.

### 2.4 Procedure

All recordings were performed in a quiet, temperature-controlled room using a NeXus-10 MKII system (Mind Media BV, Netherlands). EEG was recorded at 256 Hz using Ag/AgCl electrodes at Fp1 and Fp2 (10–20 system), with the ground at the right mastoid and bipolar referencing between Fp1 and Fp2. The electrode impedance was maintained below 10 kΩ. Data acquisition and physiological signal recording were performed using BioTrace+ software (Mind Media BV, Netherlands). Preprocessing and spectral analysis were conducted using BrainVision Analyzer (v2.01; Brain Products GmbH, Germany), applying standard filtering and semi-automatic artifact rejection procedures. Low-beta activity was defined as 12–15 Hz, while gamma band analysis was defined as 30–80 Hz. Band power (absolute or log-transformed, as exported) was calculated as the mean across the artifact-free epochs and channels. Autonomic measures were simultaneously recorded using a NeXus peripheral sensor. Blood volume pulse (BVP) signals were used to derive the heart rate (HR, beats per minute). Heart rate variability (HRV) indices included LF% (low-frequency component in normalized units, computed by the device software’s algorithm) and HRV amplitude (an amplitude-based metric, exported in arbitrary units).

### 2.5 Outcomes and statistical analyses

The outcomes comprised EEG band-power indices (gamma, low-beta activity) and autonomic measures derived from BVP (HR, HRV-LF%, HRV amplitude). Data were maintained in long format and summarized for each Group×Time cell (Pre, Post) using mean, SD, median, and 95% CIs (*n* = 3 per arm). Given the pilot sample, the analyses were descriptive and exploratory; no null hypothesis tests or between-group inferences were performed. Within-group pre–post magnitude was expressed as Hedges’ g (small-sample bias corrected) with 95% CIs, alongside the percent change to aid interpretability. Confidence intervals for cell means used t-distributions with df = n − 1; effect size estimates and CIs followed jamovi defaults. Descriptive statistics were computed using jamovi 2.6 (macOS), and figures were produced in R 4.4 via RStudio 2025.05.0 + 496 using ggplot2.

## 3 Results

### 3.1 Participants and baseline characteristics

**Figure 1:**
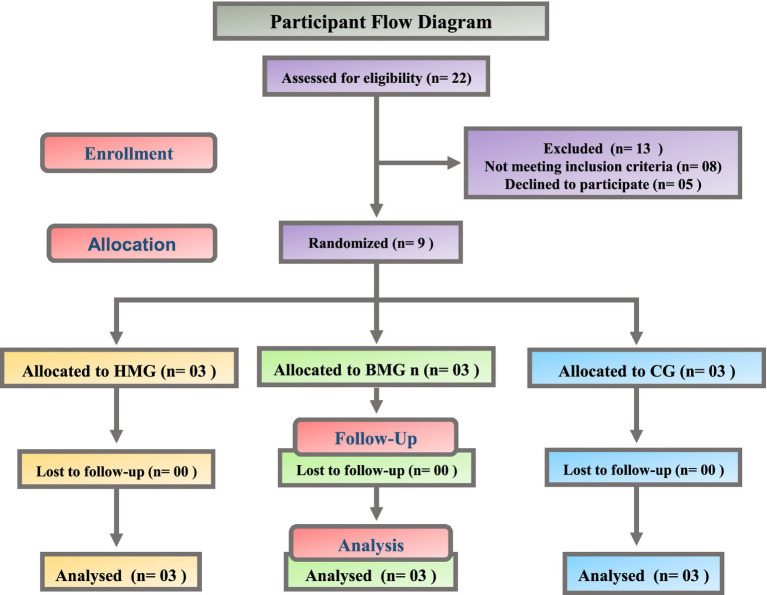
Participant flow diagram. Assessed for eligibility (*n* = 22); excluded (*n* = 13: not meeting inclusion criteria, *n* = 8; declined, *n* = 5). Randomized (*n* = 9) to HMG (*n* = 3), BMG (*n* = 3), CG (*n* = 3). No loss to follow-up; all analyzed (*n* = 3 per arm).

Of the 22 students screened, 13 were excluded (8 not meeting the inclusion criteria and five declined), leaving nine randomized to the HMG (*n* = 3), BMG (*n* = 3), or CG (*n* = 3). All completed both assessments with no losses, protocol deviations, or adverse events. The baseline characteristics were comparable across the groups as shown in Table 1. The participant flow is illustrated in Figure 1.

### 3.2 EEG outcomes

**Figure 2:**
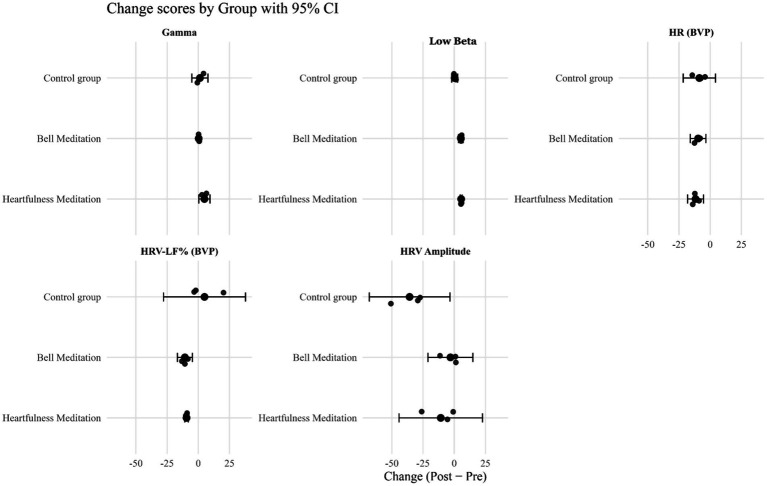
Individual pre–post trajectories for EEG and autonomic outcomes by group. The lines show individual participant values (*N* = 3 per group) for EEG gamma, low-beta activity, HR (BVP), HRV-LF%, and HRV amplitude at baseline (Pre) and post-intervention (Post). Red = Heartfulness meditation, green = Bell meditation, blue = control.

**Figure 3:**
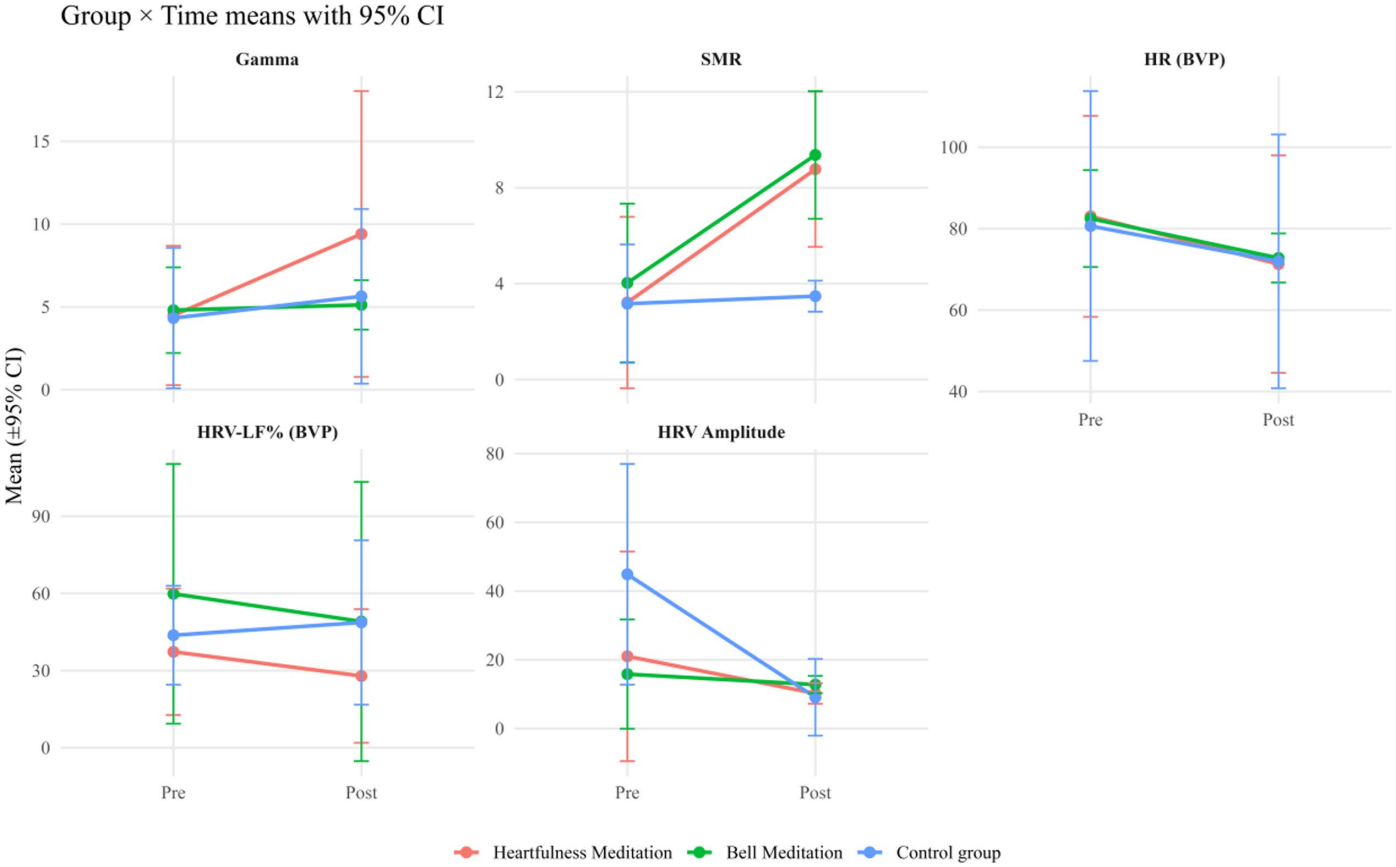
Group mean (±95% CI) changes in EEG and autonomic outcomes. Mean values with 95% confidence intervals are displayed for each group at pre- and post-intervention. The outcomes included EEG gamma, low-beta activity, HR (BVP), HRV-LF%, and HRV amplitude. Red = Heartfulness meditation, green = Bell meditation, blue = Control.

**Figure 4:**
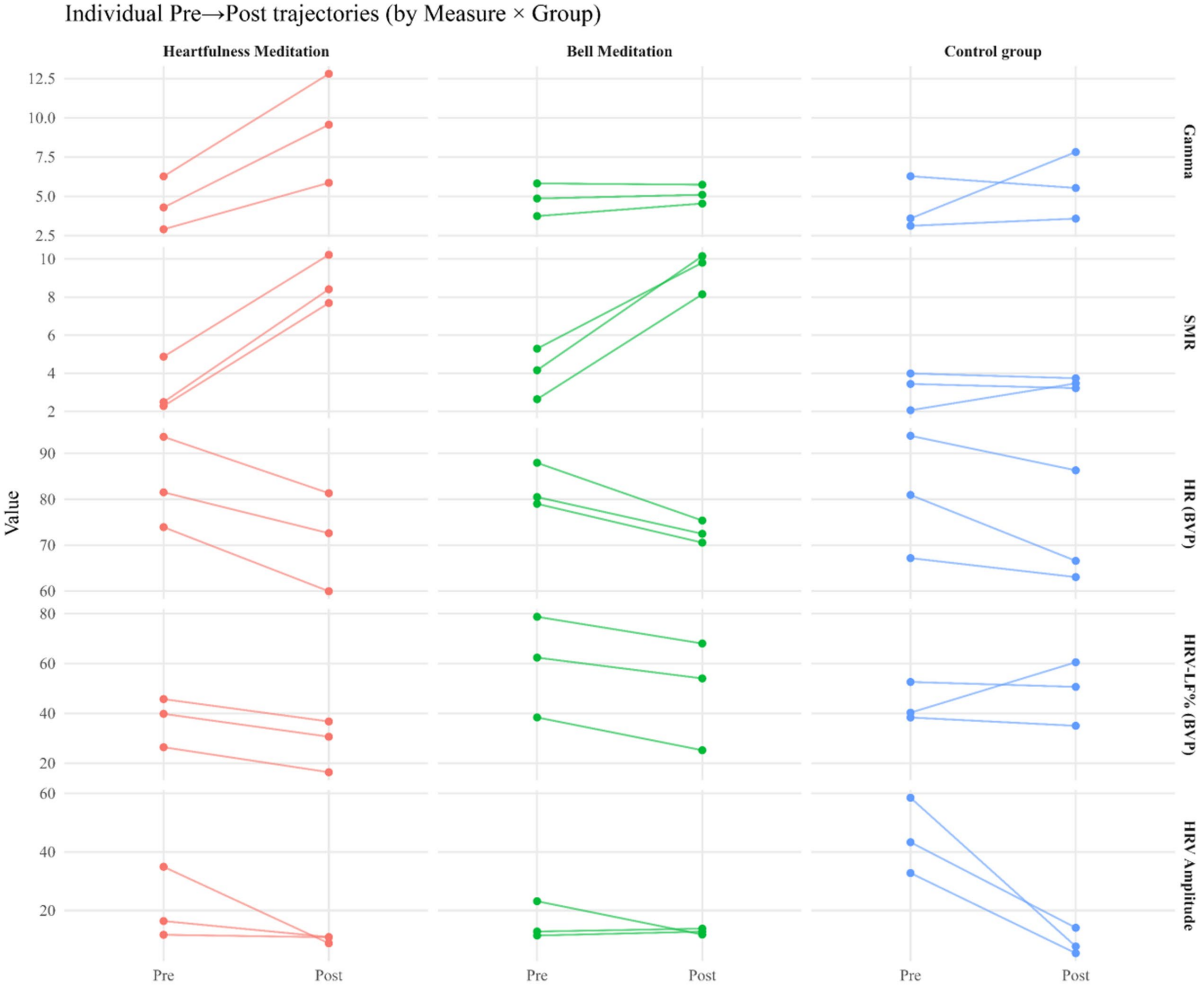
Change scores (post – pre) for EEG and autonomic outcomes by group. Box-and-whisker plots (with individual points) depict change scores for EEG gamma, low-beta activity, HR (BVP), HRV-LF%, and HRV amplitude. Groups: heartfulness meditation, bell meditation, and control. Positive values indicate increases; negative values indicate decreases from baseline.

From pre- to post-assessment, gamma power exhibited an increase of 109.6% in the heartfulness meditation group (HMG) [*g* = 1.03, CI (0.12, 1.94)], 6.6% in the bell meditation group (BMG) [*g* = 0.22, CI (−0.64, 1.08)], and 30.3% in the Control Group (CM) [*g* = 0.39, CI (−0.50, 1.28)]. Additionally, Low-Beta power increased by 173.2% in the HMG [*g* = 2.31, CI (1.07, 3.55)] and by 132.3% in the BMG [*g* = 2.52, CI (1.24, 3.80)], with a comparatively smaller change of 10.1% in the CM [*g* = 0.25, CI (−0.63, 1.13)]. Individual pre–post trajectories are depicted in Figure 2, group means with 95% confidence intervals are illustrated in Figure 3, detailed cell-wise descriptive statistics are provided in Table 2, and change score distributions are presented in Figure 4.

**Table 2:** Summary of pre–post changes in EEG and autonomic indices by group: means, percent change, and within-group effect sizes (Hedges’ g, 95% CI).

Outcome	Group	Pre mean	Post mean	Mean change	% change	Effect size (g)	95% CI lower	95% CI upper
Gamma	Heartfulness	4.49	9.41	4.92	109.6	1.03	0.12	1.94
Gamma	Bell	4.81	5.13	0.32	6.6	0.22	−0.64	1.08
Gamma	Control	4.33	5.64	1.31	30.3	0.39	−0.5	1.28
Low-Beta Activity	Heartfulness	3.21	8.77	5.56	173.2	2.31	1.07	3.55
Low-Beta Activity	Bell	4.03	9.36	5.33	132.3	2.52	1.24	3.8
Low-Beta Activity	Control	3.16	3.48	0.32	10.1	0.25	−0.63	1.13
HR (BVP)	Heartfulness	83.02	71.29	−11.73	−14.1	−0.65	−1.46	0.16
HR (BVP)	Bell	82.48	72.8	−9.68	−11.7	−1.46	−2.27	−0.65
HR (BVP)	Control	80.65	71.97	−8.68	−10.8	−0.38	−1.19	0.43
HRV-LF%	Heartfulness	37.33	27.93	−9.4	−25.2	−0.53	−1.18	0.12
HRV-LF%	Bell	59.85	49.1	−10.75	−18.0	−0.29	−0.94	0.36
HRV-LF%	Control	43.75	48.73	4.98	11.4	0.27	−0.38	0.92
HRV Amplitude	Heartfulness	21.01	10.21	−10.8	−51.4	−0.8	−2.05	0.45
HRV Amplitude	Bell	15.82	12.79	−3.03	−19.2	−0.42	−1.67	0.83
HRV Amplitude	Control	44.86	9.08	−35.78	−79.8	−1.77	−3.02	−0.52

Pre and Post denote baseline and post-intervention, respectively; Δ = Post − Pre; %Δ = 100 × Δ/Pre. Effect sizes were within-group Hedges’ g (bias-corrected) with 95% CIs; analyses were descriptive (*n* = 3 per arm). EEG band power (Gamma, Low-Beta Activity) in μV^2^; HR in bpm (BVP-derived); HRV-LF% in percent; and HRV amplitude in arbitrary units. HRV, heart-rate variability; BVP, blood-volume pulse.

### 3.3 Autonomic outcomes

These outcomes were observed from pre- to post-assessment. The heart rate, derived from BVP, decreased by 14.1% in the HMG [*g* = −0.65, CI (−1.46, 0.16)], by 11.7% in the BMG [*g* = −1.46, CI (−2.27, −0.65)], and by 10.8% in the CM [*g* = −0.38, CI (−1.19, 0.43)]. HRV-LF% showed a decline of 25.2% in HMG [*g* = −0.53, CI (−1.18, 0.12)] and 18.0% in BMG [*g* = −0.29, CI (−0.94, 0.36)], while it increased by 11.4% in CM [*g* = 0.27, CI (−0.38, 0.92)]. The HRV amplitude decreased by 51.4% in HMG [*g* = −0.80, CI (−2.05, 0.45)], by 19.2% in BMG [*g* = −0.42, CI (−1.67, 0.83)], and by 79.8% in CM [*g* = −1.77, CI (−3.02, −0.52)]. These patterns of change are illustrated in Figure 3 and Table 2, with the distribution of change scores depicted in Figure 4. Given the pilot sample size, the findings are presented descriptively rather than inferentially.

## 4 Discussion

This exploratory investigation examined changes in EEG activity and autonomic regulation following 21 days of heartfulness and bell meditation in healthy novice practitioners. Both meditation groups demonstrated increased gamma and low-beta power. Heartfulness practice produced the most pronounced effects, with a observed increase in gamma activity (*g* = 1.03) and an increase in the low-beta power (*g* = 2.31). These results support earlier evidence that even short-term meditation can enhance the high-frequency oscillations associated with certain parallel changes and sensory integration (Braboszcz et al., 2017; Aviad et al., 2024). Gamma oscillations (~30–80 Hz) are excellent markers of meditation-induced neuroplasticity (Galuske et al., 2019), reflecting integrative attentional processes (Gruber et al., 1999). The particularly strong gamma enhancement with heartfulness aligns with reports that heart-centered meditation evokes heightened gamma activity in both novice and experienced practitioners (Van’t Westeinde and Patel, 2022). The low-beta activity enhancement observed in both meditation arms suggests the facilitation of calm alertness and relaxed focus (Reichert et al., 2015). Bell meditation yielded a smaller gamma gain (*g* = 0.22) but a very notable shift in the low-beta activity (g = 2.52). This pattern is consistent with its auditory entrainment mechanism: Tibetan singing bowls are known to synchronize low-frequency theta rhythms (~6–7 Hz) (Kim et al., 2013), which promote deep relaxation and parasympathetic activation (Jirakittayakorn and Wongsawat, 2017). The relatively modest gamma effect but pronounced low-beta activity gain highlights how sound-driven practices may preferentially cultivate states of relaxation rather than any other physiological change associated with attention. By contrast, the control group (CG) exhibited only small–moderate gamma (*g* = 0.39) and small low-beta activity (*g* = 0.25) changes (Michael et al., 2005; Callan et al., 2023). Collectively, these results indicate that both meditative practices strongly promoted low-beta activity-related calm engagement, whereas heartfulness increased high-frequency activity that requires further validation with advanced artifact rejection (like ICA) activity tied to attentional integration.

Autonomic markers showed converging evidence of meditation-linked regulation. The heart rate (HR) decreased across all groups, reflecting general relaxation (Léonard et al., 2019). However, effect sizes differed: heartfulness produced a moderate reduction (*g* = −0.65), bell meditation yielded an observed decline (*g* = −1.46), and controls showed only a small–moderate change (*g* = −0.38). The low-frequency heart-rate variability (HRV) percentage (LF%) declined in both meditation arms, signifying a shift toward parasympathetic dominance: moderate in heartfulness (*g* = −0.53) and small in bell (*g* = −0.29). In contrast, the LF% increased slightly in the controls (*g* = 0.27). These findings align with prior work showing heartfulness stabilizes cardiac rhythms and reduces resting heart rate, while singing-bowl interventions enhance cardiovascular and psychological well-being (Anurag et al., 2023; Arya et al., 2018). Notably, HRV amplitude decreased in all groups, underscoring the need for more reliable indices, such as the root mean square of successive differences (RMSSD) or high-frequency power in future studies. Meta-analyses remain inconclusive regarding resting vagal HRV improvements from mindfulness-based programs, suggesting the present LF% results are preliminary but consistent with session-based vagal enhancement (Brown et al., 2021).

The parallel EEG and autonomic changes point toward early movement toward neural–autonomic physiological shifts. Specifically, the combination of observed high-frequency EEG gains with LF% reductions suggests a trend toward integrated brain–heart regulation (Natarajan, 2023). Distinct style-specific profiles emerged: heartfulness emphasized attentional/gamma engagement alongside a moderate vagal shift, while bell meditation emphasized relaxation and HR reduction with low-beta activity increases (Anurag et al., 2023). Prior studies have shown experienced heartfulness practitioners exhibit strong EEG–ECG (electrocardiography) coupling; the present findings suggest that even brief training may foster this integration (Anurag et al., 2023; Thakur et al., 2023; Krishna et al., 2025).

### 4.1 Limitations

Key limitations include a very small, male-only sample (*n* = 3/arm), reliance on descriptive analyses, a brief 21-day duration, and BVP-derived HRV and thus observed effect sizes should be treated as hypotheses for future research rather than definitive proof of efficacy. Crucially, frontal gamma findings (Fp1, Fp2) must be interpreted with extreme caution; without advanced artifact rejection (e.g., ICA), these results may be confounded by overlapping frontalis muscle tension (EMG). Additionally, resting-only measurements may obscure meditation state effects. Future trials require active controls, broader EEG montages, artifact removal, and psychological endpoints to confidently isolate the effects of these practices.

### 4.2 Future directions

Large gender-diverse samples with longitudinal tracking could clarify the dose–response relationships and trait-level changes. Continuous EEG–HRV recordings during meditation revealed dynamic physiological patterns. Comparing diverse techniques—heart-centered, sound-based, mindfulness, or movement —may delineate unique versus shared mechanisms. The integration of behavioral outcomes, such as cognition and emotion regulation, could link physiology to lived benefits. Finally, advanced methods (e.g., network connectivity and nonlinear dynamics) could be used to map how meditation cultivates adaptive neurocardiac states.

## 5 Conclusion

This pilot study provides preliminary profiles of two understudied meditation styles. In this small sample, heartfulness was associated with gamma enhancement and vagal-shifted HRV, whereas bell meditation corresponded with more modest neural changes but likely operated via auditory-driven relaxation. Both foster EEG–HRV patterns consistent with balanced arousal and resilience. Although limited, these findings generate hypotheses for controlled studies exploring how different contemplative traditions support neurocardiac health and well-being.

## Data Availability

The raw data supporting the conclusions of this article will be made available by the authors, without undue reservation.
